# Hand Furuncular Myiasis of an Infant in the Western Region of Saudi Arabia: A Case Report

**DOI:** 10.7759/cureus.33201

**Published:** 2023-01-01

**Authors:** Weaam S Magram, Hadeer Y Albakri, Adil N Althobaity, Reham M Makki, Abrar T Monaqil

**Affiliations:** 1 Division of Plastic Surgery, Department of Surgery, King Abdulaziz University, Jeddah, SAU; 2 College of Medicine, King Saud bin Abdulaziz University for Health Sciences, Jeddah, SAU; 3 College of Medicine, King Abdullah International Medical Research Center, Jeddah, SAU; 4 Plastic and Reconstructive Surgery, King Abdulaziz Medical City, Jeddah National Hospital, Jeddah, SAU

**Keywords:** dermatobia hominis, western region of saudi arabia, cordylobia anthropophaga, furuncular myiasis, cutaenous myiasis

## Abstract

Furuncular myiasis is a rare disease that affects the skin and is caused by growing maggots of different types of fly species within the arthropod order Diptera. The symptoms of the disease include itching, a sensation of movement, and sometimes fever. The disease predominantly occurs in tropical and subtropical areas. In Saudi Arabia, furuncular myiasis is reported to occur frequently in the Western region. Herein, we present a case of a 10 months-old Saudi girl who came with multiple lesions over her scalp and left hand starting five days following a trip to Al Shafa, southwest of Saudi Arabia. The patient’s lesion was red, solid, and increased in size gradually. On examination, a papule with a central punctum was present on the left hand at the dorsal aspect of the first web space. The patient underwent an urgent operation to extract the larvae under general anesthesia. Excision of the furuncular myiasis larvae was done using a punch-biopsy blade with pressurized irrigation of the pocket with normal saline and diluted betadine solution. After two weeks, the patient showed a completely recovered skin infection. Having sufficient clinical awareness is necessary to prevent such disease, diagnose it, and prevent further spreading.

## Introduction

Furuncular myiasis develops after the skin layer has been infested by the growing maggots of numerous fly species belonging to the arthropod order Diptera [1]. Dermatobia hominis and Cordylobia anthropophaga are the two fly species that most frequently infest people worldwide [1]. A variety of factors predispose humans to be susceptible to furuncular myiasis, including poor social conditions and hygiene, and the presence of chronic diseases [2]. The infestations are usually found in South and Central America, and the most common age group described is 41 to 50 years old [2].

The typical symptoms of furuncular myiasis include itching, a sensation of movement inside the lesion, and stabbing or sharp pain [3]. The typical manifestation of furuncular lesions includes an erythematous, furuncle-like papule, with one or more larvae inside it [4]. The papule contains a central punctum that exudes purulent or serosanguinous fluid [4]. Inside the central pore, the parasite's presence can be seen by simply visualizing the lesion [4].

## Case presentation

Herein, we present a case of a 10-month-old Saudi girl who was healthy and up-to-date with the vaccines. She presented to the emergency department of National Guard Hospital, Jeddah, Saudi Arabia, with multiple lesions over her scalp and left hand starting five days following a trip to Al-Shafa, southwest of Saudi Arabia. The patient was playing there with soil, and the mother said there were a lot of mosquitoes in that place.

The mother noticed the lesion first in the left hand of her baby and took her to a nearby clinic where she was diagnosed with a possible bacterial abscess and was prescribed sodium fusidate ointment and discharged. The following day, the mother noticed similar lesions on her daughter’s scalp. The mother could clearly visualize the larvae's tail coming out of the central punctum of the lesion. As described by the mother, the lesions were red, solid, and gradually increasing in size. Moreover, there was a history of difficulty sleeping, decreased appetite, crying, and itching. On the other hand, there was no history of fever, and the history of other systems involvement was absent. Also, there were no similar lesions in other family members.

**Figure 1:**
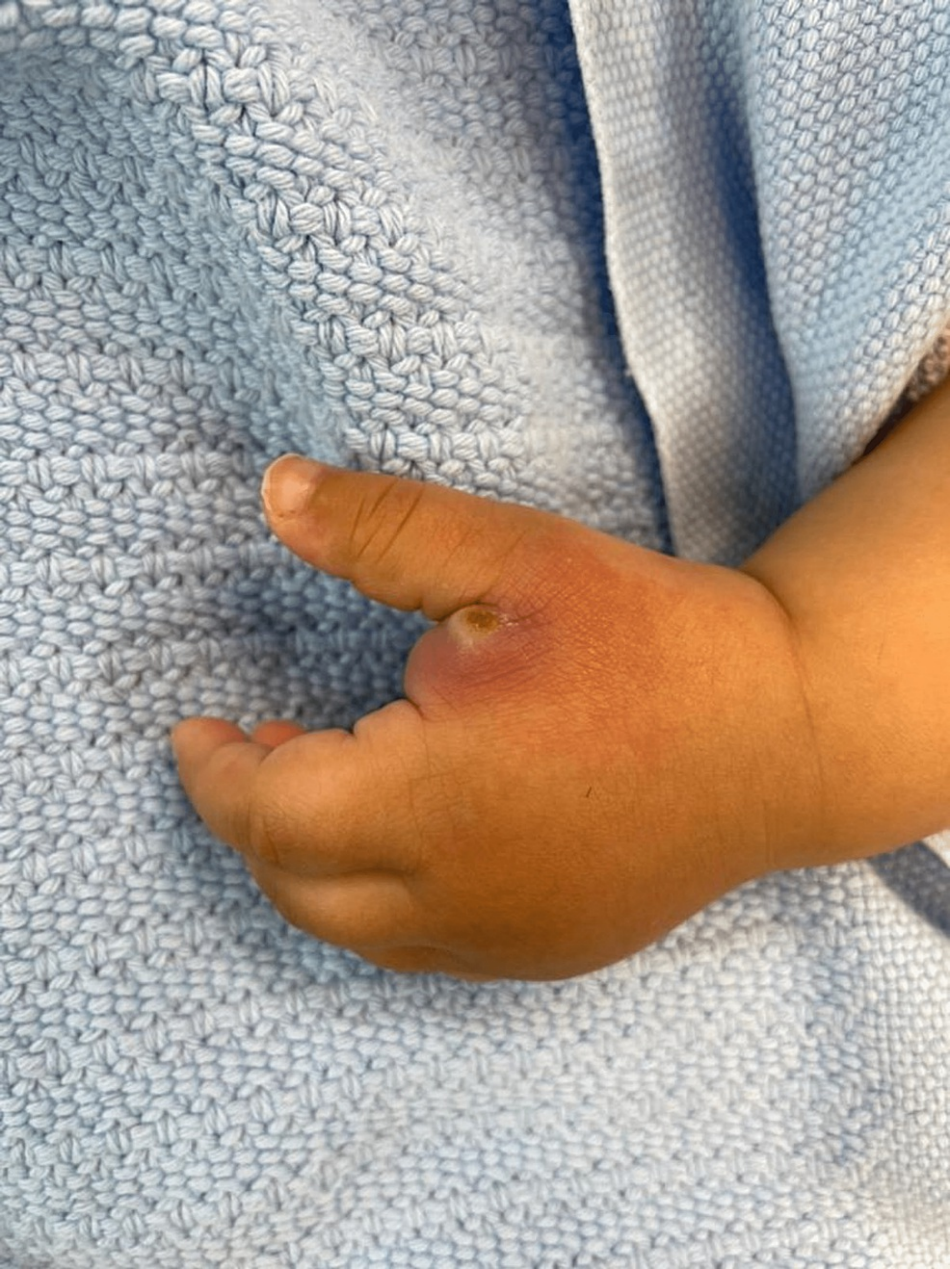
A 10-month-old baby’s left hand with a typical furuncular myiasis lesion

On examination, the patient looked healthy and was not irritable. She was vitally stable with blood pressure 101/60 mmHg, heart rate 130 Freq/min, respiratory rate 30 Freq/min, temperature 36.6 celsius, and oxygen saturation 100% on room air. A papule with a central punctum was present on the left hand at the dorsal aspect of the first web space with a swelling involving the dorsal and volar aspect of the first web space (Figure 1). The patient was vitally stable with blood pressure 101/60 mmHg, heart rate 130 Freq/min, respiratory rate 30 Freq/min, temperature 36.6 celsius, and oxygen saturation 100% on room air.

**Figure 2:**
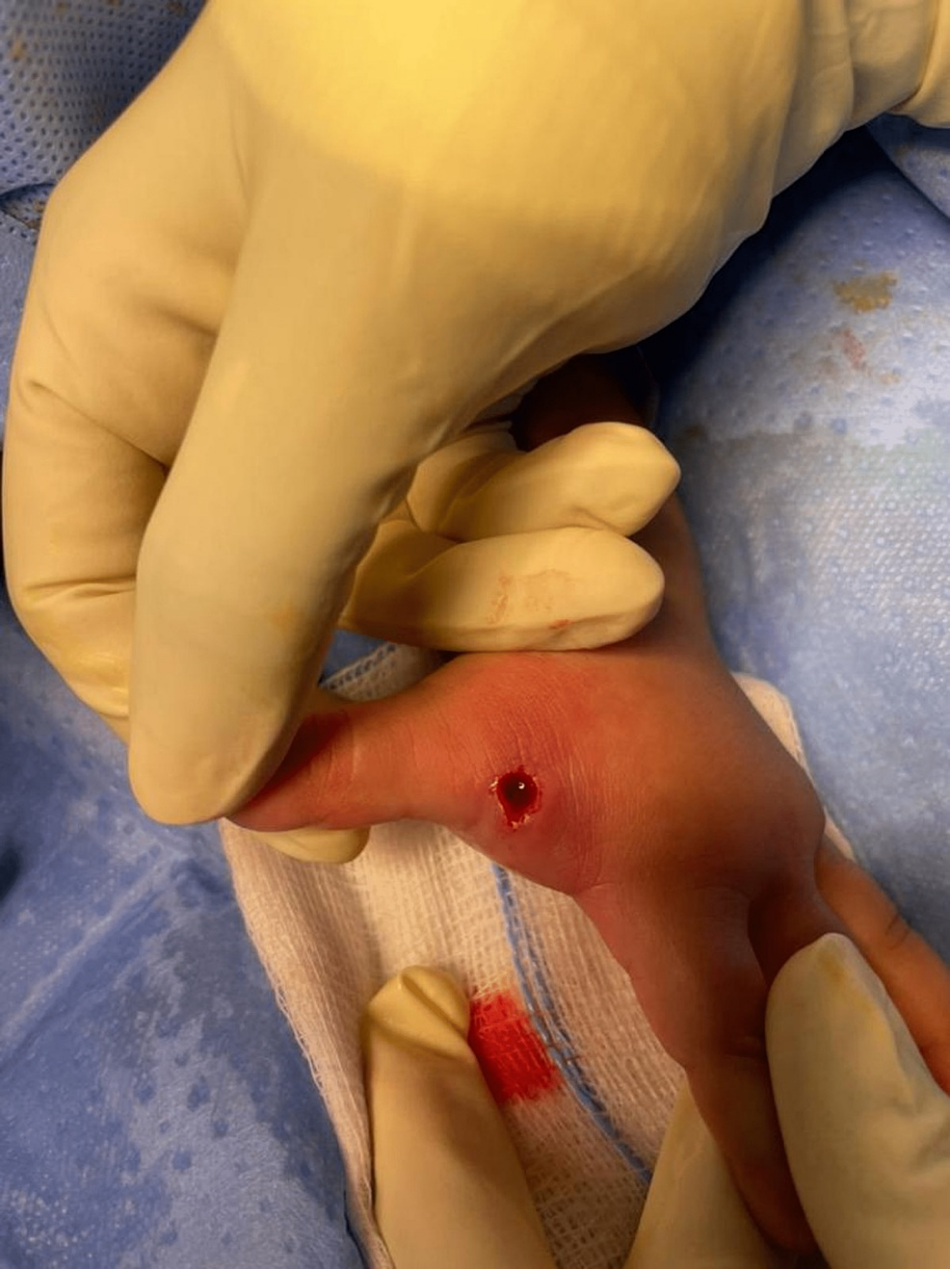
The lesion after furuncular myiasis larvae were extracted using a punch-biopsy blade

**Figure 3:**
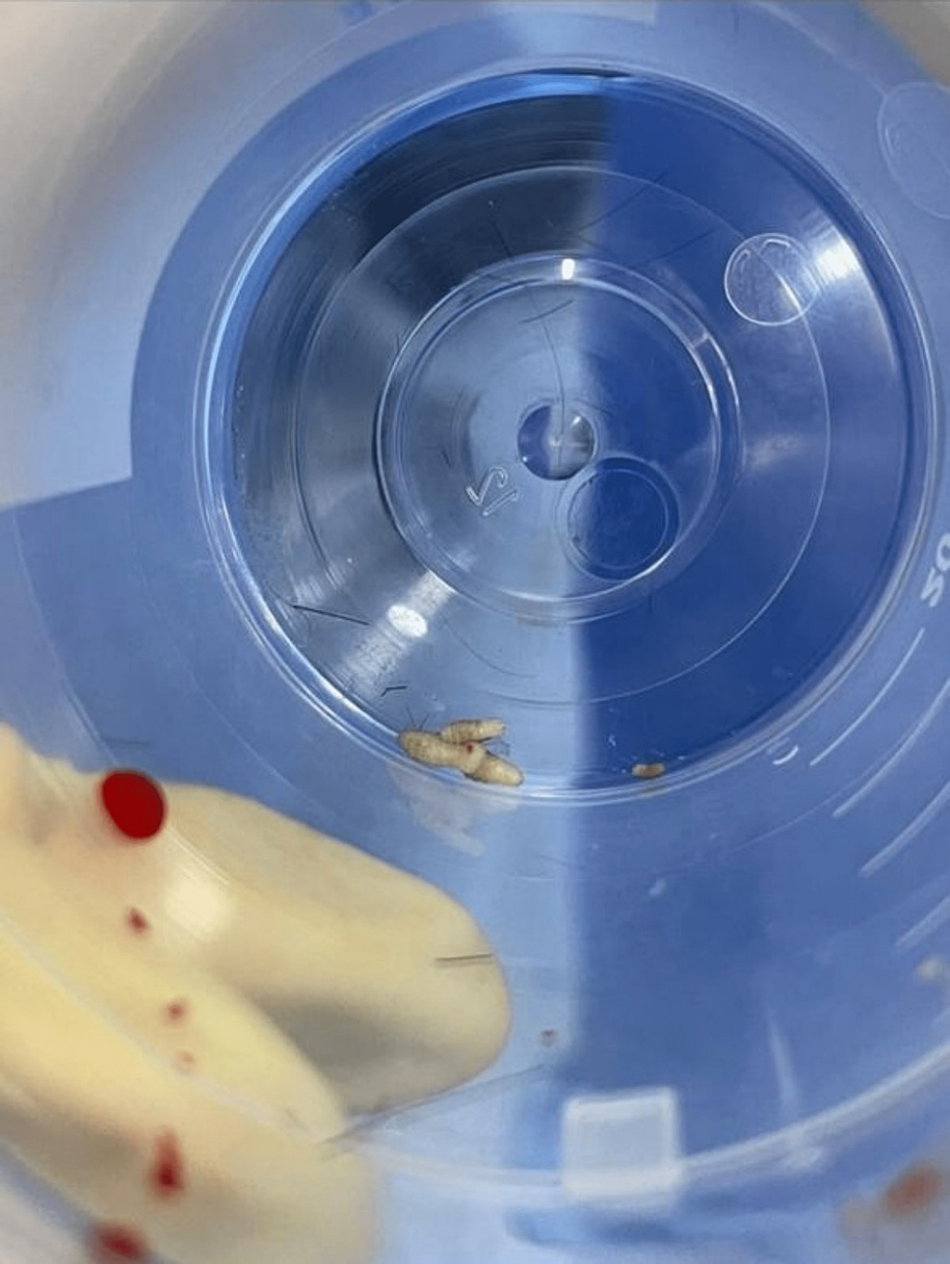
The parasites (Dermatobia hominis) after being extracted

The hand was warm, erythematous, and tender. However, the patient had no abscess fluctuation, pus discharge, regional adenopathy, or signs of lymphangitis. Nervous system examination was normal. Also, there were similar lesions present on the scalp of the patient. Laboratory parameters showed normal white blood cells and inflammatory markers. A decision was made to extract the larvae surgically under general anesthesia. The patient was prepared to undergo an operation to extract the larvae under general anesthesia. The patient was admitted under the care of pediatric surgery and we, as a plastic surgery team, were consulted regarding the hand infestation. The patient was given antibiotics to prevent secondary bacterial infection. However, they did not recommend starting the patient on any antihelminthic medication. In the theater, the patient was placed in a supine position, and prepping and draping of the left hand in a standard sterile manner was done. After that, the removal of the maggot was done with a punch biopsy tool (Figure 2 and Figure 3).

**Figure 4:**
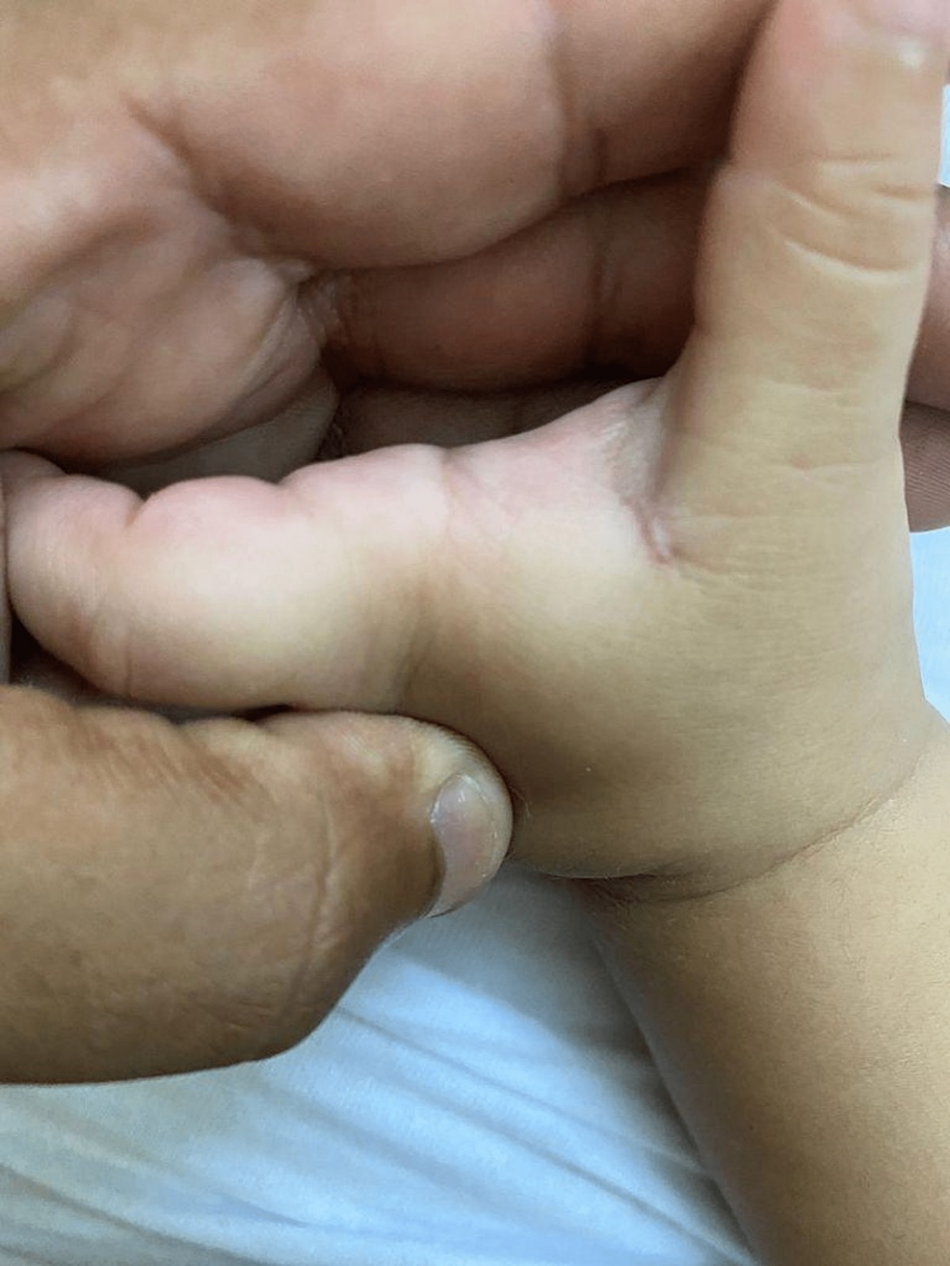
The furuncular myiasis lesion fully healed (after two weeks)

Then, pressurized irrigation of the pocket with 300 ml of normal saline and 20 ml of diluted betadine solution (antimicrobial) was done. Finally, we packed the area, and dressing was applied. The specimen was sent to another histopathology lab in King Abdulaziz Medical City, Riyadh, and was verified as Dermatobia hominis by a dermatopathologist consultant. Also, in regard to scalp lesions, the pediatric surgery team joined us and removed the larvae by incision and drainage. On review after two weeks, there was complete resolution of the skin lesion (Figure 4).

## Discussion

Myiasis is a seasonal infection [1]. In Saudi Arabia, furuncular myiasis is reported to occur most frequently in Asir and Al-Baha (Western Saudi Arabia) and rarely occurs in other regions of Saudi Arabia [5]. Also, furuncular myiasis is much more frequent in the tropics and subtropics of the United States and Africa [1]. The latitude and life cycles of the different fly species influence the prevalence [1]. In temperate zones, the flies are only present during the summer; however, they are present all year round in tropical regions since they prefer a warm, humid atmosphere [1].

Furuncular myiasis is a rare human disease [1]. A few cases reported in Saudi Arabia are similar to our case. Mohammed AA et al. reported a case of a 10-year-old girl who presented to King Abdulaziz University Hospital in 2013, complaining of two boil-like nodular lesions on the back of the neck and the left axilla since three weeks after a vacation on a farm in Al-Baha city, Southwestern Saudi Arabia. The lesion was pruritic papules then developed into painful nodules after a few days. The patient did not have a fever, however, she had a sense of malaise. The case was managed by removing the larvae using forceps and gentle squeezing [6]. Gabra AO et al. reported a one-year-old boy who presented to a pediatric surgery clinic one week after visiting Altaif, Saudi Arabia, complaining of an itchy, painful nodule on the inner aspect of his right forearm. The nodule had serous discharge in the center of the punctum. The mother of the child denied any spikes of fever, changes in activity, or decreased oral intake. The lesion was managed at the bedside by squeezing the punctum after dilating it using curved artery forceps with no antibiotics prescribed [7].

**Figure 5:**
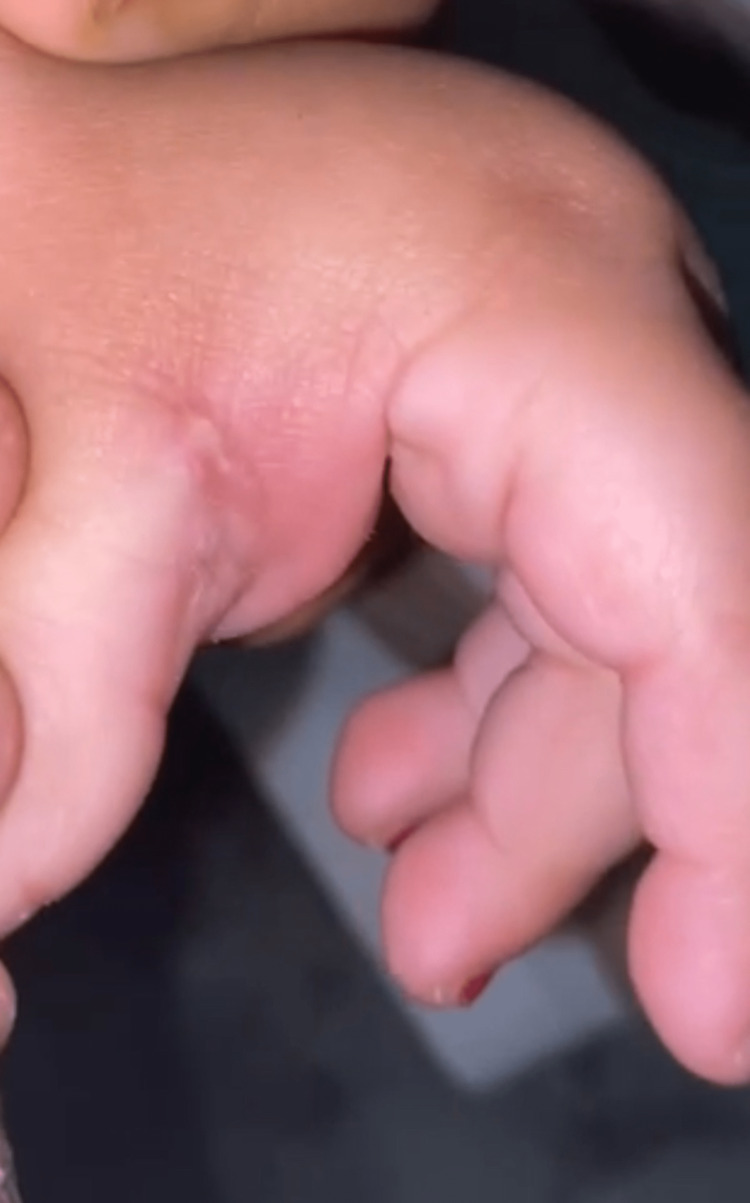
The fully healed furuncular myiasis lesion (after six months)

Different modalities of management of furuncular myiasis have been described in the literature, starting from simple extraction of the larva and expanding to excision of the bed of larvae [1,2]. The two previous cases that have been mentioned above were mostly managed by manual removal. However, in our case, we utilized the punch biopsy tool to extract the bed of the larvae, as it is justified that manual extraction alone might leave some remnant of the hooks inside the wound and cause further irritation and granulomatous inflammation. Moreover, punch biopsy excision resulted in a good prognosis and minimal scar formation. As the lesion is an apparent, functional, and aesthetic area, we aimed for effective management by preventing the contractures or deformities of the first web space. Punch biopsy decreases contracture formation as it creates a circular defect that limits the length of the scar compared with a linear scar after elliptical excision. Upon follow-up at six months, the patient showed clearance of the furuncular myiasis without recurrence and complete healing of the lesion (Figure 5).

## Conclusions

In conclusion, the presented case highlights that clinical awareness and learning methods for preventing furuncular myiasis are needed to minimize its spread. Furuncular myiasis must be identified early by history and physical examination to provide appropriate management and avoid complications. Additionally, we encourage utilizing the punch biopsy tool in excising the furuncular myiasis lesions, as it ensures clearing the bed of any remnants of hooks or eggs and heals with minimal scarring and contractures.
